# N-3 Fatty Acids (EPA and DHA) and Cardiovascular Health - Updated Review of Mechanisms and Clinical Outcomes

**DOI:** 10.1007/s11883-025-01363-2

**Published:** 2025-11-17

**Authors:** Ivana Djuricic, Philip C. Calder

**Affiliations:** 1 Department of Bromatology, Faculty of Pharmacy, University of Belgrade, Belgrade, Serbia; 2 School of Human Development and Health, Faculty of Medicine, University of Southampton, IDS Building, MP887 Southampton General Hospital, Tremona Road, Southampton, SO16 6YD UK; 3 NIHR Southampton Biomedical Research Centre, University Hospital Southampton NHS Foundation Trust and University of Southampton, Southampton, UK

**Keywords:** N-3 PUFAs, Fish oil, Cardiovascular disease, Heart disease, Atherosclerosis, Risk factor

## Abstract

**Purpose of Review:**

We synthesize the latest evidence (published 2020 to 2025) on the role of eicosapentaenoic acid (EPA) and docosahexaenoic acid (DHA) in cardiovascular health, emphasizing biological mechanisms and key findings from observational studies and clinical trials related to cardiovascular disease (CVD) risk and outcomes.

**Recent Findings:**

EPA and DHA modulate lipid metabolism, inflammation, platelet and endothelial function, the gut-heart axis, ion channels and autonomic function via vagal tone, supporting cardiovascular health. While individual RCTs have produced variable results, updated cohort data and recent meta-analyses consistently link higher intake or circulating levels of EPA and DHA to reduced risk of cardiovascular events. However, evidence from RCTs indicates that high-dose supplementation may be associated with an increase in atrial fibrillation (AF) risk.

**Summary:**

Evidence supports a role for EPA and DHA in CVD prevention and treatment, with effects influenced by dose, formulation, and individual variability. Moderate intake appears safe and protective, while high dose EPA may offer added benefits in high-risk individuals but also might increase AF risk.

## Introduction

Cardiovascular disease (CVD) continues to be a leading cause of morbidity and mortality worldwide, even though global CVD mortality decreased by ~ 35% from 1990 to 2022 [1]. Among the numerous dietary factors investigated, n-3 polyunsaturated fatty acids (n-3 PUFAs), particularly eicosapentaenoic acid (EPA) and docosahexaenoic acid (DHA), have been extensively explored for their potential cardioprotective and other health effects [2–6]. Marine microalgae are the major producers of EPA and DHA; hence some species of algae and their oils are good sources of these fatty acids. EPA and DHA pass through the marine food chain, meaning that seafood in general is a rich source of EPA and DHA; oily (fatty) fish are the richest dietary source of EPA and DHA. For example, fatty fish such as mackerel, salmon, trout, herring, pilchards and sardines typically provide between 1.5 and 3 g EPA + DHA per serving, with the precise amount depending upon the exact species, the season and location of the catch, and whether the fish is wild or farmed [7]. Lean fish, such as cod, plaice and haddock typically provide 200 to 400 mg EPA + DHA per serving; crustaceans such as crab, prawns and shrimp may provide 60 to 850 mg EPA + DHA per serving depending upon the exact source; and shellfish such as mussels may provide 200 to 300 mg EPA + DHA per serving. Oil extracted from the flesh of different fish species or from the liver of some species (such as cod) contains EPA and DHA, which typically comprise about 30% of the fatty acids present in “fish oils”; these oils are the basis for production of more highly concentrated EPA and DHA rich pharmaceutical grade oils. Some crustaceans such as krill and other marine organisms such as calanus are also sources of oils containing EPA and DHA. The meat and fat of sea mammals such as whale and seal are also rich in EPA and DHA, but are rarely eaten by humans, apart from in certain cultures. In contrast to seafood, the meat of terrestrial animals is relatively low in EPA and DHA. For example, lamb, beef, pork and poultry meat typically provide 20 to 50 mg of EPA + DHA per serving. One hen’s egg can provide 30 to 70 mg of DHA. However, there is evidence that altered feeding practices can modestly increase the content of EPA and DHA in terrestrial meats, milks and eggs [8]. Plants produce a different n-3 fatty acid, α-linolenic acid. While this is a metabolic precursor to EPA, and perhaps also DHA, in humans [9], plants themselves do not produce EPA and DHA. Therefore, while some plant sources such as flaxseeds and flaxseed oil, chia seeds, walnuts and soybean oil provide α-linolenic acid, they are not sources of EPA and DHA [10].

Analysis for the Global Burden of Disease Study 2017, identified that a diet “low in seafood n-3 fatty acids” (i.e. EPA and DHA) was the sixth leading dietary risk factor for both mortality and disability-adjusted life-years, especially those due to CVD, globally and in many countries, being a greater risk than diets low in fibre or high in trans fats or meat, although a lower risk than diets high in sodium, low in whole grains, low in fruits, low in nuts and seeds or low in vegetables [11].

The “omega-3 index” (n-3 index) has been established as a standard biomarker for assessing an individual’s or population’s EPA and DHA status and as a risk factor for death from coronary heart disease [12]. The n-3 index represents the percentage contribution of EPA and DHA to all fatty acids in red blood cell membranes; other measures of blood EPA + DHA levels such as in whole blood, plasma or plasma phospholipids can be converted into an estimated n-3 index [13], with levels categorized as desirable (>8%), moderate (>6% to 8%), low (>4% to 6%), or very low (≤ 4%). The global n-3 PUFA maps from 2016 and the updated 2024 version [13] based on n-3 index reveal how EPA + DHA status in many countries is low or very low but that this has changed across some countries over time. Some countries, such as Japan and Norway, continue to maintain high levels (i.e. desirable n-3 index) due to diets rich in fatty fish, while others, like USA and parts of Europe remain low or very low but some show modest improvements, likely influenced by increased use of EPA and DHA supplements and improved dietary awareness [13]. A recent study from 7 countries (USA, Canada, Spain, Italy, Germany, Japan and South Korea) reported that only cohorts from Alaska, South Korea and Japan achieved a desirable n-3 index [14]. Although some countries showed moderate increases in EPA and DHA levels, the overall global status of these n-3 PUFAs remains suboptimal, with many populations still at risk of low status [13, 14] and of high rates of non-communicable disease, especially CVD [1, 11]. This review aims to present the most recent evidence (papers published since 2020 but integrated with key earlier findings) on how EPA and DHA contribute to cardiovascular health, including updated insights into their biological mechanisms and findings from clinical outcomes related to CVD.

## Mechanisms of Action of EPA and DHA Related To Decreased CVD Risk and Mortality

EPA and DHA promote several biological actions that contribute to their cardiovascular protective effects. These effects include modulation of lipid metabolism; anti-inflammatory, antioxidant, antiarrhythmic and antithrombotic effects; cell membrane effects including modulation of ion channel function and of formation of membrane domains involved in cell signalling; and endothelial function improvement [2–6].

### Improved Lipid Metabolism

The well-documented triglyceride (TG)-lowering effects of EPA and DHA [15] are primarily attributed to enhanced hepatic fatty acid oxidation, decreased *de novo* lipogenesis and synthesis of very-low-density lipoproteins (VLDLs), and improved clearance of TG-rich lipoproteins (TGRLs) such as chylomicrons and VLDLs, thereby shortening their circulation time [16]. Cell culture and animal studies have shown that these effects are partly mediated by the activation of the peroxisome proliferator activated receptor (PPAR) α, which plays a key role in regulating expression of genes related to lipid metabolism [17]. Additionally, EPA and DHA reduce the delivery of non-esterified fatty acids to the liver and promote the formation of phospholipids over TGs [18]. Further, studies have identified another mechanism involving the formation of N-acyl taurines (NATs) derived from EPA and DHA, which accumulate in bile and plasma when these fatty acids are present. In particular, DHA-derived NAT has been shown to inhibit intestinal TG hydrolysis and lipid absorption, contributing to lower plasma TG levels and reduced hepatic fat accumulation in experimental animals [19]. This mechanism may partly explain DHA’s slightly greater efficacy over EPA in reducing plasma TG concentrations [19, 20]. While both EPA and DHA effectively lower TG levels, they exert distinct effects on lipoprotein subparticles, i.e., EPA tends to decrease HDL3, whereas DHA increases HDL2 [21, 22]. HDL2 has been generally considered more protective against CVD than HDL3 due to its larger size and its role in reverse cholesterol transport. While HDL3 can also provide some protection, the benefit is less pronounced compared to HDL2 [23]. DHA, but not EPA may also modestly raise LDL-cholesterol levels and increase LDL particle size, without altering apolipoprotein B (Apo-B) concentrations, suggesting a shift toward less atherogenic LDL particles (see [24] and references therein). In contrast, EPA exhibits unique antioxidant properties, likely due to its optimal structure; an in vitro study showed that EPA may inhibit LDL oxidation and reduce cholesterol domain formation within membranes, enhancing lipoprotein clearance and diminishing atherogenicity [25]. Moreover, biophysical research highlights the differing impacts of EPA and DHA on membrane structure: EPA appears to maintain phospholipid organization and an even distribution of cholesterol, whereas DHA disrupts membrane order, leading to cholesterol aggregation [26, 27].

### Anti-Inflammatory Effects

Alongside atherogenic lipid modulating effects, EPA and DHA impact acute and chronic inflammation reducing levels of circulating inflammatory markers such as interleukin (IL)−6, IL-1β and tumor necrosis factor-alpha (TNF-α) [28, 29]. Cell culture and animal studies support that EPA and DHA reduce inflammation-related gene activity by inhibiting the nuclear factor kappa beta (NF-κB) signalling pathway, either by preventing IκB phosphorylation or activating nuclear receptors PPARα and γ [30]. Additionally, EPA, docosapentaenoic acid (DPA) and DHA are precursors of specialized pro-resolving mediators (SPMs), including resolvins, protectins, and maresins of different kinds which do not merely suppress inflammation, but actively initiate resolution of inflammation and tissue repair [31]. These effects are relevant to the impact of EPA and DHA on CVD since inflammation is an important contributor to growth of the atherosclerotic plaque and to its rupture. Indeed, the ratio of DHA-derived resolvin D1 to arachidonic acid (AA)-derived leukotriene B_4_, a pro-inflammatory mediator, predicted carotid intima-media thickness among 254 individuals with mean age 66 years [32]. Human trials have shown incorporation of EPA and DHA into human carotid atherosclerotic plaques with a period of supplementation (approx. 1.8 g EPA + DHA/day for several weeks) and that this is associated with decreased plaque inflammation [33, 34] and increased plaque stability [33].

More recently, attention has been paid to whether EPA and DHA modulate the gut microbiome and, in turn, influence cardiovascular risk [35]. Bidirectional communication between the gut and cardiac system creates a gut-heart axis. Research suggests that EPA and DHA can modulate gut microbiota composition, particularly affecting the ratio of *Firmicutes* (F) to *Bacteroidetes* (B); an increase in the F/B ratio may lead to overweight, obesity, non-alcoholic fatty liver disease, and CVD (see [4] and references therein). By helping restore the F/B ratio, EPA and DHA are linked to increased production of the anti-inflammatory short-chain fatty acid butyrate, which has been shown to lower the expression of inflammatory mediators [35]. Maintaining eubiosis contributes to improved gut barrier function. A less resilient intestinal barrier permits the translocation of bacterial endotoxins, lipopolysaccharides (LPS) and microorganisms into the bloodstream, a process known as a “leaky gut” [36]. For instance, when LPS enters the circulation, it can initiate systemic inflammation, which plays a critical role in promoting atherosclerosis and other cardiovascular pathologies [37, 38]. Cell culture and animal studies revealed that EPA and DHA exert potent anti-inflammatory effects by inhibiting NF-κB signalling pathways activated by LPS in monocytes, macrophages, dendritic cells and endothelial cells [39]. In macrophages, EPA and DHA both attenuate LPS-induced mitogen-activated protein kinase (MAPK) activity and suppress the production of pro-inflammatory mediators such as TNF-α [40]. Additionally, they promote the release of anti-inflammatory cytokines such as IL-10 from resident macrophages. They also facilitate the expansion of regulatory T cells (Tregs) while restraining the proliferation of T helper 17 (Th17) cells [41]. Given that Th17 cells produce IL-17, a cytokine implicated in tissue inflammation, the modulation of the Th17/Treg balance by EPA and DHA may contribute to reduced inflammatory responses both systemically and in the gut [42].

### Antiarrhythmic Effects

**Fig. 1:**
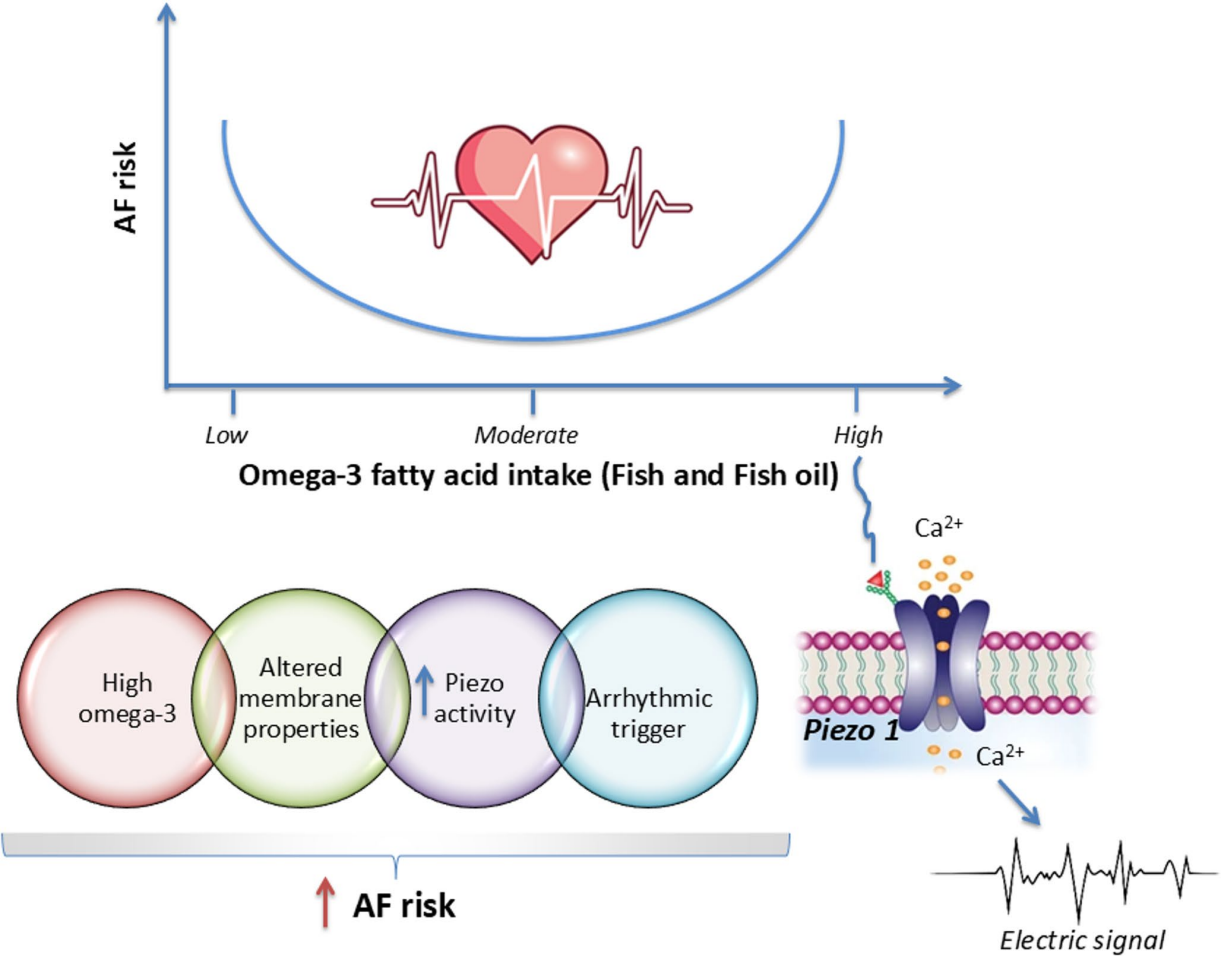
U-shaped relationship between EPA and DHA intake and risk of atrial fibrillation (AF). Moderate doses offer cardioprotection, while high doses may increase AF risk. High −n-3 PUFA intake may influence PIEZO1 channel activity, affecting calcium influx and triggering delayed after-depolarizations and AF

The antiarrhythmic effects of EPA and DHA result from multiple complementary mechanisms, including modulation of ion channels and influence on the autonomic nervous system. EPA and DHA improve cardiac muscle cell function and alter membrane fluidity, affecting ion channel conductivity and reducing heart rate [43]. However, by directly influencing calcium, sodium, and potassium currents in the heart, they can also change the duration of ventricular action potentials. As a result, EPA and DHA, while generally anti-arrhythmic, may under certain conditions promote re-entrant arrhythmias. Recent studies suggest that PIEZO proteins mediate the effects of EPA and DHA on atrial fibrillation (AF) [44]. PIEZO1/2 are among the largest known ion channels, comprising over 2,500 amino acids arranged into 38 transmembrane helices. Cell membrane lipids modulate the structural transitions that control the opening and closing of these channels. It has been shown that DHA slows PIEZO1 inactivation, while EPA accelerates it. Therefore, the DHA and EPA ratio might influence PIEZO1 activity [44]. A predominance of DHA enhances PIEZO1-mediated calcium influx, prolonging the action potential and raising the risk of delayed after-depolarizations and AF. It also promotes calcium-dependent signalling pathways. PIEZO1 is not limited to cardiomyocytes; atrial fibroblasts also express this channel. Notably, fibroblasts from patients with AF show higher PIEZO1 expression and activity compared to those with normal sinus rhythm, implicating it in atrial structural remodelling [45]. Beyond ion channels, PIEZO1-related alterations may affect ionic pumps, membrane receptors, and cytoskeletal interactions, collectively contributing to a pro-arrhythmic environment. EPA and/or DHA-induced modulation of PIEZO1 is thus one of several membrane-level changes that may increase AF susceptibility (Fig. 1) [46].

Abundant in the membranes of cardiomyocytes and neurons, EPA and DHA help regulate autonomic activity by enhancing vagal tone, reflected in lower resting heart rate, increased heart rate variability (HRV), and faster post-exercise heart rate recovery [47]. On the other hand, high EPA and DHA dose mediated vagal nerve stimulation appears to be a risk for developing AF [47].

### Antithrombotic Effects and Endothelial Function

EPA and DHA are incorporated into platelet membrane phospholipids in place of the n-6 fatty acid AA. This alters the substrate available for cyclooxygenase, resulting in increased production of thromboxane (TX) A₃ and prostaglandin (PG) I₃ from EPA rather than TXA₂ and PGI₂ (often called prostacyclin) from AA. TXA₃ has markedly weaker platelet-activating effects than TXA₂, while PGI₃ retains the vasodilatory and anti-aggregatory activity of PGI₂ [48]. In endothelial cells, n-3 PUFAs stimulate NO production by activating endothelial NO synthase (eNOS) through both Ca²⁺-independent pathways and caveolin-1 dissociation from eNOS, as well as by activating TRPV4 Ca²⁺ channels [49]. These mechanisms, together with reduced inflammation and platelet aggregation, enhance flow-mediated vasodilation and contribute to improved endothelial function and lower cardiovascular risk [50]. In addition to inhibiting platelet aggregation [51] and enhancing endothelial function [52, 53], the combination of EPA and DHA has been shown to modulate the biogenesis, molecular composition and prothrombotic activity of extracellular vesicles (EVs), especially platelet-derived EVs (PDEVs), which represent the predominant EV subtype in the circulation and contribute significantly to endothelial dysfunction, thrombosis, and the progression of CVD. PDEVs enhance coagulation by exposing phosphatidylserine and supporting thrombin generation, making them highly thrombogenic, up to 100 times more than activated platelets [54].

## Evidence from large-scale Cohort Studies and Pooled Analyses

In recent years, the relationship between EPA and DHA and CVD has continued to be a focus of epidemiological research, mainly through large-scale prospective cohort studies; an earlier systematic review and meta-analysis (published in 2014) identified that those in the highest tertile of dietary intake of EPA + DHA or in the highest tertile of blood levels of EPA, DPA, DHA or EPA + DHA were at lower risk of coronary outcomes over the follow-up period than those in the lowest tertiles [55]. Using data from 17 prospective cohort studies, Alexander et al. [56] reported an 18% lower risk for any coronary heart disease event for subjects with higher dietary intake of EPA + DHA than for those with lower intake with a median follow-up of 12.5 years. There were also significant reductions of 19, 23, and 47% in the risk for fatal coronary death, coronary events, and sudden cardiac death, respectively. Pooling data from 19 cohorts showed that EPA and DHA were each independently associated with a reduction in the risk of fatal coronary heart disease, with about a 10% reduced risk for each one standard-deviation increase in either EPA or DHA measured in a blood pool or in adipose tissue [57]. A comprehensive pooled analysis published in 2021 examined the association between blood levels of different n-3 PUFAs and mortality outcomes [58]. This study synthesized data from 17 prospective cohorts, encompassing 42,466 individuals with a median follow-up of 16 years. Participants in the highest quintile of circulating EPA, DPA, DHA or EPA + DHA had a lower risk of death from all causes (15 to 18%) or from CVD (13 to 21%) than those in the lowest quintile.

A much larger pooled analysis published in 2024 and involving over 183,000 participants from 29 international prospective cohorts investigated the association between blood levels of EPA and DHA and the risk of incident stroke with a median follow-up of 14.3 years [59]. Those in the highest quintile of circulating levels of EPA or DHA had significantly lower risk of total and ischemic stroke than those in the lowest quintile, while neutral association was reported for hemorrhagic stroke (no increased risk). Participants with the highest quintile of EPA levels had a 17% lower risk of total stroke (Hazard Ratio (HR): 0.83; 95% confidence interval (CI): 0.76–0.91) and an 18% lower risk of ischemic stroke (HR: 0.82; 95% CI: 0.74–0.91) compared to those in the lowest quintile. Similarly, those in the highest quintile of DHA showed a 12% reduced risk of total stroke (HR: 0.88; 95% CI: 0.81–0.96) and a 14% lower risk of ischemic stroke (HR: 0.86; 95% CI: 0.78–0.95). The protective associations were consistent across various subgroups, including individuals with and without pre-existing cardiovascular conditions or AF.

A prospective cohort study by Chiusolo et al. [60] published in 2023 explored whether the ratio of selected n-3 to n-6 PUFAs in adipose tissue is a better predictor of myocardial infarction (MI) than the levels of selected n-3 PUFAs alone. Drawing from the Danish Diet, Cancer and Health Cohort, which included over 57,000 participants, subcutaneous adipose tissue samples were analyzed from 3,500 individuals. These participants were then followed for 15 years to monitor the occurrence of MI. The study found that both the absolute levels of EPA, DPA and DHA and their ratios to AA (such as EPA/AA, DHA/AA, and combined EPA + DPA + DHA/AA) were inversely associated with the risk of MI, but ratios provided superior risk prediction.

Another pooled analysis, published in 2024, examined whether EPA and DHA intake modifies the CVD risk associated with a family history of CVD [61]. The study analyzed data from 40,885 CVD-free adults across 15 observational cohorts, assessing blood and tissue levels of EPA and DHA. A significant interaction was observed between low levels of EPA/DHA (≤ 25th percentile) and a family history of CVD. The combined exposure (low EPA + DHA and family history) was associated with a 41% higher risk of incident CVD (Risk Ratio (RR): 1.41; 95%, CI: 1.30–1.54) compared to individuals with neither exposure. A family history of CVD alone was associated with a 25% increased risk (RR: 1.25; 95% CI: 1.16–1.33), while low EPA + DHA levels alone showed a 6% increase in risk (RR: 1.06; 95% CI: 0.98–1.14).

A study published in 2023 focussed to cardiac rhythm disorders, specifically AF [62]. This study, comprising over 54,000 participants from 17 cohorts, assessed blood levels of EPA, DPA, DHA, and EPA + DHA combined over a median follow-up of 13.3 years. While EPA alone showed no association with increased risk of incident AF, higher levels of DPA, DHA, and EPA + DHA were modestly associated with a lower risk of AF (HR: 0.89 (95% CI: 0.83–0.95), HR: 0.90 (95% CI: 0.85–0.96), HR: 0.93 (95% CI: 0.87–0.99), respectively). Further clarity comes from the Million Veteran Program, a large-scale cohort study of over 300,000 US veterans also published in 2023 [63]. This study examined dietary intake of EPA, DPA and DHA and their relationship to incident AF. Higher dietary intake was associated with a lower risk of developing AF: the median intake of EPA + DHA + DPA was 219 mg/d (IQR: 144–575) and the protective effect was most notable up to an intake of approximately 750 mg/day (11% reduction in AF risk at this intake), indicating a threshold beyond which additional intake conferred no extra benefit. Similarly, in 2025 O’Keefe et al. [47] pooled data from 17 prospective cohorts and 54,799 participants and found that higher blood levels or dietary intake of EPA and DHA, were generally associated with a lower risk of developing AF. The maximal risk reduction, approximately 12%, was observed at an intake of around 650 mg/day of EPA + DHA.

Using UK Biobank data, several studies have investigated the relationship between n-3 PUFA biomarkers or intake and cardiovascular risk. One large prospective analysis based on this resource followed more than 415,000 individuals aged 40–69 years over 12 years [64]. It was found that among participants without existing CVD, regular use of fish oil supplements, a source of EPA and DHA, was associated with an increased risk of developing AF (HR: 1.13, 95% CI: 1.10–1.17) and a slightly elevated risk of stroke (HR: 1.05, 95% CI: 1.00–1.11). However, among those with established CVD, fish oil use was beneficial, lowering the risk of progression from AF to major adverse cardiovascular events (MACE) (HR 0.92, 95% CI: 0.87–0.98), MI (HR 0.85, 95% CI: 0.76–0.96), and death (HR: 0.91, 95% CI: 0.84–0.99). An additional observational study using UK Biobank data assessed AF risk among participants without pre-existing AF, including 261,108 individuals with measured plasma n-3 PUFA levels and 466,169 who reported whether they used fish oil supplements or not [65]. Over a median follow-up of 12.7 years, higher plasma total n-3 PUFAs or DHA or “non-DHA n-3 PUFAs” (mainly EPA) were all associated with a lower risk of developing AF, with those in the highest quintile of plasma DHA having an 8% lower risk compared to those in the lowest quintile (HR: 0.92; 95% CI: 0.87–0.97) and those in the highest quintile of plasma “non-DHA n-3 PUFAs” having an 16% lower risk compared to those in the lowest quintile (HR: 0.84; 95% CI: 0.80–0.88). Fish oil supplement use was reported by 31% of participants, more commonly among older adults. However, after adjusting for age as a continuous variable, fish oil supplement use showed no association with AF risk (HR: 1.00; 95% CI: 0.97–1.02). Complementing these results, another study conducted within the UK Biobank further underscored the cardiovascular benefits of n-3 PUFAs, this time in relation to heart failure (HF) [66]. In a cohort of 271,794 participants without HF at baseline, a linear inverse association was observed between plasma total n-3 PUFA, DHA and “non-DHA n-3 PUFAs” (mainly EPA) levels and incident HF over a median follow-up of 13.7 years. Participants in the highest quintile of n-3 PUFA levels had a 21% lower risk of developing HF compared to those in the lowest quintile (HR: 0.79; 95% CI, 0.74–0.84; *P* < 0.001). Similarly, among individuals with preexisting HF at baseline, higher n-3 PUFA (or DHA or “non-DHA n-3 PUFAs”) levels were associated with markedly lower risks of both all-cause mortality (HR: 0.53; 95% CI, 0.33–0.86) and cardiovascular mortality (HR: 0.50; 95% CI, 0.31–0.79).

## Evidence from major RCTs reporting on cardiovascular outcomes

Over the years, a large number of RCTs have investigated the effects of EPA and DHA in CVD primary and secondary prevention and treatment, as reviewed in detail elsewhere [3, 6]. However, results have not always been consistent; while earlier trials often reported significant protective effects, more recent large-scale RCTs have mostly shown more modest or even neutral outcomes (Table 1).

**Table 1:** Summary of the main randomized controlled trials of EPA and DHA on cardiovascular outcomes

Trial and year of publication	Patient group	Number of patients	EPA + DHA dose (mg/day)	Form of EPA and DHA	Control	Average duration of follow-up (yr)	Main findings with EPA and DHA
GISSI-Prevenzione 1999 [67]	Recent MI	11,334	460 + 380	Ethyl ester	None	3.5	Reduced both primary outcomes (composite of death, non-fatal MI or non-fatal stroke (−15%) and composite of cardiovascular death, non-fatal MI or non-fatal stroke (−20%)) and secondary outcomes (all fatal events (−20%); cardiovascular death (−30%); cardiac death (−35%); coronary death (−35%); sudden death (−45%))
JELIS 2007 [73]	Hypercholesterolemia (primary prevention) or Hypercholesterolemia + pre-existing CHD (secondary prevention)	18,645	1800 + 0 (+ statin)	Ethyl ester	Statin alone	4.6	Primary prevention: No effect Secondary prevention: Fewer non-fatal coronary events (−19%)
OMEGA 2010 [68]	Recent MI	3851	460 + 380	Ethyl ester	Olive oil	1.0	No effect on primary outcome (sudden death) or secondary outcomes (mortality; composite of mortality, MI or stroke; revascularization)
Alpha-Omega 2010 [69]	Previous MI	4837	226 + 150 (in margarine)	Triglyceride	Standard margarine	3.4	No effect on primary outcome (composite of fatal or non-fatal CVD or need for cardiac intervention) or secondary outcomes (mortality; fatal CVD; fatal CHD; ventricular arrhythmia-related evets). Reduced fatal CHD and fewer arrhythmia-related events in patients with diabetes.
ORIGIN 2012 [70]	Dysglycemia plus recent MI or heart failure	12,536	460 + 380 (*±* long-acting insulin)	Ethyl ester	Long-acting insulin or standard care alone	6.2	No effect on primary outcome (death from cardiovascular causes), secondary outcomes (mortality; death from arrhythmia; composite of death from cardiovascular causes, non-fatal MI or non-fatal stroke) or other outcomes (MI; stroke; revascularization; hospitalization for heart failure)
ASCEND 2018 [71]	Diabetes	15,480	460 + 380	Ethyl ester	Olive oil	7.4	No effect on primary outcome (serious vascular events: composite of non-fatal MI, non-fatal stroke, transient ischemic attack or vascular death), secondary outcome (serious vascular events or revascularization) or components of primary outcome except fewer vascular deaths (−19%)
REDUCE-IT 2019 [72]	CVD risk and raised triglycerides (primary prevention) or Established CVD and diabetes and raised triglycerides (secondary prevention); all on statins	8179	3600 + 0	Ethyl ester	Mineral oil	4.9	Reduction in primary outcome (composite of cardiovascular death, non-fatal MI, non-fatal stroke, revascularization or unstable angina) (−25%)), secondary outcome (composite of cardiovascular death, non-fatal MI or non-fatal stroke) (−20%)) and multiple other outcomes (cardiovascular death or non-fatal MI (−25%); MI (−31%); revascularization (−35%); cardiovascular death (−20%); hospitalization for angina (−32%); stroke (−28%); death, non-fatal MI or non-fatal stroke (−23%))
STRENGTH 2020 [74]	CVD risk and raised triglycerides; all on statins	13,078	2200 + 800	Free fatty acid	Corn oil	3.5	No effect on primary outcome (composite of cardiovascular death, non-fatal MI, non-fatal stroke, revascularization or unstable angina) or any secondary outcome (composites of outcomes; cardiovascular death; mortality)
OMEMI 2021 [77]	Recent MI	1027	930 + 660	Triglyceride	Corn oil	2	No effect on primary outcome (composite of non-fatal MI, stroke, mortality, revascularization or heart failure) or on any component of primary outcome; no effect on secondary outcome (new onset atrial fibrillation although that tended to be higher with n-3 PUFAs)
OMEGA REMODEL 2024 [78]	Recent MI	358	1860 + 1500 (6 months)	Ethyl ester	Corn oil	6.6	No effect on primary outcome (composite of all-cause death, heart failure hospitalizations, recurrent acute coronary syndrome, late coronary artery bypass graft)
RESPECT-EPA 2024 [80]	Stable CAD and EPA/AA < 0.4; all on statins	3800	1800 + 0	Ethyl ester	None	5	No effect on primary outcome (cardiovascular death, non-fatal MI, non-fatal ischemic stroke, unstable angina pectoris, coronary revascularization); Significant reduction in secondary outcome (composite of sudden cardiac death, MI, unstable angina, and coronary revascularization (−27%)); new onset atrial fibrillation seen in EPA group
VITAL 2019 [81] Bayesian reanalysis of VITAL 2025 [82]	Healthy (> 50 years old)	25,871	460 + 380 (*±* vitamin D)	Ethyl ester	“Matching placebo”	5.3	No effect on primary outcome (composite of MI, stroke or death from cardiovascular causes), secondary outcome (primary outcome or revascularization) or some individual components of these outcomes (stroke; death from CHD; death from CVD; death from stroke) but reduction in MI (−28%), CHD (−17%) and death from MI (−50%). Bayesian analyses confirmed the robustness of n-3 PUFA effects on CAD and MI (HRs 0.88–0.93 and 0.82–0.90; >99% probability of benefit). Modest reductions were seen in CVD, cardiovascular, and all-cause death (HRs ~ 0.91–0.96; ≥98% probability). Stroke risk was unchanged (33.7% probability of benefit).

*EPA* eicosapentaenoic acid, *DHA* docosahexaenoic acid, *MI* myocardial infarction, *CVD* cardiovascular disease, *CHD* Coronary Heart Disease, *CAD* Coronary Artery Disease

One of the earliest and most influential studies, the GISSI-Prevenzione trial published in 1999, demonstrated a significant reduction in cardiovascular death, including sudden cardiac death, in post-MI patients who received 840 mg/day of EPA + DHA for 3.5 years [67]. Although it was an open-label study with no placebo, this trial laid the foundation for EPA and DHA therapy in secondary prevention. In contrast, subsequent trials such as OMEGA [68] and Alpha-Omega [69], both randomized, placebo-controlled, double-blind trials published in 2010, failed to replicate these benefits. These trials used similar or lower doses of EPA + DHA in post-MI populations but found no significant impact on cardiovascular outcomes. These trials were characterized by shorter follow-up durations than GISSI-Prevenzione and potentially insufficient EPA and DHA dosing. The ORIGIN (double-blind) (published in 2012) [70] and ASCEND (placebo-controlled) (published in 2018) [71] trials investigated supplementation with 840 mg/day EPA + DHA among patients with dysglycemia and diabetes, respectively, and also found no decrease in major cardiovascular events. A turning point came with the REDUCE-IT, a randomized, placebo-controlled, double-blind trial published in 2019, which used a high dose (3.6 g/day) of purified EPA as an ethyl ester (referred to as icosapent ethyl) in statin-treated patients with elevated triglycerides and established CVD or diabetes [72]. This EPA formulation had been used at a lower dose (1.8 g/day) in combination with statins in an earlier Japanese trial (published in 2007) with positive cardiovascular outcomes compared with stains alone [73]. REDUCE-IT reported a 25% decrease in MACE, including reductions in MI, stroke, and cardiovascular death. REDUCE-IT stands out as the only new trial showing strong cardiovascular benefits, likely due to its high EPA dose and use of a purified EPA formulation. In comparison, STRENGTH, a randomized, placebo-controlled, double-blind trial published in 2020 tested a similar high dose (2.2 g EPA and 0.8 g DHA daily) but of a mixed EPA + DHA as free fatty acids [74]. STRENGTH was terminated early due to futility, as no cardiovascular benefit was observed. The divergent outcomes between REDUCE-IT and STRENGTH have raised interest in understanding whether DHA may counteract EPA’s benefits or whether the formulation and placebo choice played a role [75]. REDUCE-IT used a mineral oil placebo, which some argue may have had pro-inflammatory or LDL-cholesterol-raising effects, potentially exaggerating the apparent efficacy of EPA [76]. Additional smaller-scale studies, such as OMEMI (published in 2021), which targeted an elderly post-MI population and utilized a moderate EPA + DHA dose (1.6 g/day), also failed to demonstrate cardiovascular benefit [77]. All these studies are comprehensively previously discussed [3].

The pilot multicenter, double-blind, placebo-controlled OMEGA-REMODEL trial published in 2024 evaluated the long-term effects of EPA + DHA on adverse cardiac events following acute MI [78]. In this study, patients with acute MI were randomly assigned to receive either EPA + DHA (3.36 g/day) or placebo for six months. The primary endpoint was a composite of MACE, including all-cause mortality, hospitalization for heart failure, recurrent acute coronary syndrome, and late coronary artery bypass grafting. A total of 358 patients were followed for a median of 6.6 years. During follow-up, 36.1% of the EPA + DHA group and 34.8% of the placebo group experienced MACE. Intention-to-treat analysis showed no significant difference in overall MACE risk between the groups (HR: 1.014; 95% CI: 0.716–1.436; *p* = 0.938). However, about one-third of patients showed a strong biological response to treatment, defined by an increase in their n-3 index (≥ 5%) after 6 months. This subgroup had a significantly lower MACE rate (2.9%; 95% CI: 1.2–5.1) than non-responders (7.1%; 95% CI: 5.7–8.9; *p* = 0.001). The same trial reported that those patients who achieved the highest quartile increase in n-3 index experienced a 13% reduction in left ventricular systolic volume index in comparison with the lowest quartile [79].

The large, multicenter, randomized, open-label, blinded end-point RESPECT-EPA trial published in 2024 and conducted across Japan, aimed to investigate whether EPA (as icosapent ethyl) could reduce cardiovascular events in patients with stable CAD who were already on statin therapy and had low plasma levels of EPA relative to AA [80]. Over 3,800 participants were enrolled, and about 2,500 with particularly low plasma EPA/AA ratios (< 0.4) were randomly assigned to receive either daily EPA treatment (1.8 g/day) or no additional intervention. The study followed patients for a median of 5 years, tracking major cardiovascular outcomes such as MI, stroke, and revascularization procedures. While the EPA group showed a lower incidence of these events, i.e., 9.1% compared to 12.6% in the control group, the difference did not reach statistical significance (HR: 0.79; 95% CI: 0.62–1.00; *p* = 0.055). However, when looking at a secondary composite of coronary events (which included sudden cardiac death, MI, unstable angina, and coronary revascularization), the benefit of EPA treatment became more evident, with significantly fewer cases in the EPA group, 6.6% vs. 9.7%, (HR: 0.73; 95% CI: 0.55–0.97, *p* = 0.031). Importantly, while overall adverse effects were similar between groups, a higher rate of new-onset AF was observed among those receiving EPA (3.1% vs. 1.6%; *p* = 0.017).

The VITAL randomized, placebo-controlled trial (published in 2019) and the Bayesian reanalysis of VITAL (published in 2025) provide complementary perspectives on the role of EPA and DHA in cardiovascular prevention [81, 82]. While both publications examine the same dataset, they differ in their statistical approach, which influences the interpretation of the findings. The VITAL trial is the largest and only trial that investigated the primary prevention effects of EPA + DHA (840 mg/d of EPA + DHA) on CVD in a general healthy population aged over 50 years [81]. With 25,871 participants and a median follow-up of 5.3 years, the study found no statistically significant reduction in the primary outcome, major cardiovascular events (HR: 0.92; 95% CI: 0.80–1.06) or in the expanded composite endpoint (HR: 0.93; 95% CI: 0.82–1.04) [81]. There was also no effect on stroke or death from CVD. However, there were reductions in MI (HR: 0.72; 95% CI: 0.59–0.90), death from MI (HR: 0.50; 95% CI: 0.26–0.97) and developing CHD (HR: 0.83; 95% CI 0.71–0.97) [81]. In subgroup analysis, the cardiovascular benefits of EPA + DHA supplementation were most evident among participants with low baseline fish intake (< 1.5 servings per week), who experienced a 19% reduction in major cardiovascular events and a 40% reduction in MI, with the strongest effect seen in those eating fish less than once per month. Race and ethnicity also influenced outcomes: African American participants showed markedly greater reductions in MI, coronary revascularization, and total CHD compared with other groups. Reduction in MI among African Americans was observed regardless of their baseline fish intake, while among non-Hispanic whites, supplementation reduced MI only in those with low fish consumption. Notably, the baseline plasma n-3 levels did not modify the intervention’s effects, and the findings remained consistent after accounting for nonadherence [81].

The Bayesian reanalysis of VITAL incorporates prior evidence from earlier RCTs on EPA and DHA supplementation [82]. This method uses a Bayesian approach to estimate posterior probabilities for the effect of EPA + DHA supplementation on various outcomes, allowing for more nuanced conclusions. The reanalysis used informative priors, drawn from previous trials, and applied Bayesian statistical models to estimate the likelihood of beneficial effects. The key results of the Bayesian analysis show a high probability that EPA + DHA supplementation is effective in reducing the risk of CAD and MI, with posterior HRs ranging from 0.82 to 0.93 for MI and 0.88 to 0.93 for CAD. The probability of EPA + DHA effectiveness for these outcomes was high, 99.6% for MI and 99.7% for CAD, suggesting that, while the reductions in risk may be modest, the benefits are high and statistically very likely. The Bayesian analysis also supported EPA + DHA supplementation for reducing cardiovascular death and all-cause mortality, with posterior HRs of 0.91–0.92 for CVD death and 0.95–0.96 for all-cause death, both of which had probabilities of benefit above 98%. These findings align with the idea that EPA and DHA could have broader protective effects, even if the benefits in individual CVD events like stroke were less pronounced. However, the Bayesian analysis found no effect for stroke, with only a 33.7% probability that EPA + DHA supplementation would reduce stroke risk.

## Recent small-scale RCTs

A study published in 2024 by Bozbas et al. investigated the effects of EPA + DHA supplementation on extracellular vesicles (EVs) and their role in coagulation [54]. Forty participants at moderate cardiovascular risk received either fish oil (providing 1.9 g/day EPA + DHA) or high-oleic safflower oil as placebo for 12 weeks. The study found that EPA + DHA decreased circulating EV numbers by 27%, increased their n-3 PUFA content two-fold, and decreased their capacity to support thrombin generation by over 20%. However, these changes did not affect thrombus formation in ex vivo assays. These findings suggest that EPA and DHA can modulate EV characteristics and reduce their procoagulant activity, potentially contributing to CVD prevention.

In 2025, Ibrahim et al. reported the effects of EPA + DHA supplementation on heart rate variability (HRV) in 60 children aged 5 to 12 years with overweight or obesity [83]. Over three months, children who received 400 mg EPA + 200 mg DHA daily along with lifestyle advice showed significant improvements in HRV markers, i.e., RMSSD, SDNN, and pNN50 (*p* = 0.017, 0.009, and 0.043, respectively), indicating better autonomic (parasympathetic) function. They also had lower TG levels and higher HDL-cholesterol (*p* = 0.006 and 0.005, respectively). These findings suggest EPA + DHA may support cardiovascular health in this vulnerable population.

## An Update of Meta-Analyses of RCTs on EPA and DHA and Cardiovascular Outcomes

Over the years, multiple meta-analyses of the effects of EPA and DHA on CVD outcomes and mortality have been published, with varying findings, in part due to the exact studies included, as discussed in detail elsewhere [3, 6]. Table 2 summarizes findings from more recent meta-analyses in this field. A 2019 meta-analysis published soon after REDUCE-IT included data from 13 RCTs, including GISSI-Prevenzione, JELIS, Omega, Alpha Omega, ORIGIN, VITAL, ASCEND and REDUCE-IT [84]. Trials had to have a sample size of at least 500 patients and a follow-up for at least one year to be included. Total sample size of the combined trials was 127,477, with a mean duration of follow-up of 5 years. In an analysis excluding REDUCE-IT, n-3 PUFA supplementation (EPA + DHA or EPA alone) was associated with a significantly lower risk of MI (Rate Ratio (RateR): 0.88; 95% CI: 0.83–0.94; *p* < 0.001), CHD death (RateR: 0.92; 95% CI: 0.86–0.98; *p* = 0.014), CVD death (RateR: 0.92; 95% CI: 0.88–0.97; *p* = 0.003), total CVD (RateR: 0.95; 95% CI: 0.82–0.98; *p* < 0.001) and major vascular events (RateR: 0.95; 95% CI: 0.93–0.98; *p* < 0.001). These effects were all strengthened when the findings from REDUCE-IT were included. With continuing interest and new trials in the period since REDUCE-IT was published, there have been a number of new meta-analyses. A 2021 meta-analysis of 38 RCTs involving 149,051 patients evaluated the effects of EPA and DHA on fatal and non-fatal cardiovascular outcomes and the variability in EPA + DHA vs. EPA alone treatment effects [85]. Of all studies, 34 used EPA + DHA with dose ranges of 0.4 to 5.5 g/day, while 4 trials used EPA alone at 1.8 or 3.6 g/day. Effect sizes are reported as risk differences (RDs) and RateRs. This meta-analysis found that EPA and DHA were associated with reduced cardiovascular mortality (0.93; 95% CI: 0.88–0.98; *p* = 0.01), non-fatal MI (0.87; 95% CI: 0.81–0.93; *p* = 0.0001), CHD events (0.91; 95% CI: 0.87–0.96; *p* = 0.0002), MACE (0.95; 95% CI: 0.92–0.98; *p* = 0.002) and revascularization (0.91; 95% CI: 0.87–0.95; *p* = 0.0001). EPA monotherapy showed greater reduction than EPA + DHA for CV mortality (EPA: 0.82; 95% CI: 0.68–0.99 and EPA + DHA: 0.94; 95% CI: 0.89–0.99), non-fatal MI (EPA: 0.72; 95% CI: 0.62–0.84 and EPA + DHA: 0.92; 95% CI: 0.85–1.00), CHD events (EPA: 0.73; 95% CI: 0.62–0.85 and EPA + DHA: 0.94; 95% CI: 0.89–0.99), as well for MACE and revascularization. EPA and DHA increased incident AF (1.26; 95% CI: 1.08–1.48). EPA alone vs. control was associated with a higher risk of total bleeding (1.49; 95% CI: 1.20–1.84) and AF (1.35; 95% CI: 1.10–1.66).

**Table 2:** Recent meta-analyses of the effect of EPA and DHA on cardiovascular outcomes

Study and publication year	Major Outcomes	Study Design	Intervention arm	Duration of Treatment	Pooled effects of EPA and DHA vs. Placebo
Hu et al., 2019 [84]	MI, CHD death, total CHD, total stroke, CVD death, total CVD, major vascular event	Meta-analysis of 13 RCTs (from 1999 to 2018) including 127,477 participants in various states of health	0.376–4 g/d EPA + DHA or EPA alone	1–7.4 y	Significant reduction in MI (RateR: 0.88; 95% CI: 0.83–0.94), CHD death (RateR: 0.92; 95% CI: 0.86–0.98), CVD death (RateR: 0.92; 95% CI: 0.88–0.97), total CVD (RateR: 0.95; 95% CI: 0.82–0.98) and major vascular events (RateR: 0.95; 95% CI: 0.93–0.98).
Khan et al., 2021 [85]	Cardiovascular mortality, non-fatal cardiovascular outcomes, bleeding, and AF	Meta-analysis of 38 RCTs (from 1990 to 2020) including 149,051 participants in various states of health	0.4–5.5 g/d EPA + DHA or EPA alone	1–6.2 y	Significant reduction in cardiovascular mortality (RR:0.93; 95% CI: 0.88–0.98), non-fatal MI (RR: 0.87; 95% CI: 0.81–0.93), CHD (RR: 0.91; 0.87–0.96), MACE (RR: 0.95; 95% CI: 0.92–0.98), and revascularization (RR: 0.91; 95% CI: 0.87–0.95). Higher reductions with EPA monotherapy than with EPA + DHA. N-3 PUFAs increased incident AF (RR: 1.26; 1.08–1.48). EPA monotherapy increased risk of total bleeding (RR: 1.49; 95% CI: 1.20–1.84) and AF (RR: 1.35; 95% CI: 1.10–1.66).
Huang et al., 2023 [86]	Cardiovascular events (cardiovascular death or hospitalization for cardiovascular causes, fatal and/or nonfatal MI, angina, fatal and/or nonfatal stroke, heart failure, unplanned revascularization, and AF) and all-cause mortality	Meta-analysis of 8 RCTs (from 2007 to 2022) including 57,754 patients with diabetes	0.84–4 g/d EPA + DHA or EPA alone	3.5–7.4 y	Significant reduction in cardiovascular events (RR: 0.93; 95% CI: 0.90–0.97) and no effects on the risk of fatal/nonfatal MI, major vascular events, CAD, AF, or all-cause mortality. EPA alone significantly reduced the risk of CVD (RR: 0.81; 95% CI: 0.73–0.90).
Dinu et al., 2024 [87]	Incident coronary revascularizations, MI, stroke, HF, unstable angina, and cardiovascular death	Meta-analysis of 18 RCTs (from 1996 to 2023) including 134,144 participants in various states of health	0.6–5.4 g/d EPA + DHA or EPA alone	4.5 months − 7.4 y	Significant reduction in the risk of revascularization (RR: 0.90; 95% CI: 0.84–0.98), MI (RR: 0.89; 95% CI: 0.81–0.98), and cardiovascular death (RR: 0.92; 95% CI: 0.85–0.99). EPA alone was more effective than combined EPA + DHA in reducing the risk of revascularization (RR: 0.76; 95% CI: 0.65–0.88) and showed greater benefit across all outcomes except HF.
Javaid et al., 2024 [91]	Bleeding	Meta-analysis of 11 RCTs (from 2007 to 2020) including 120,643 participants in various states of health	0.84–4 g EPA + DHA or EPA alone	2–7.4.4 y	No effect on overall bleeding risk. Relative bleeding risk increased with high doses of EPA (50%), while absolute risk remained low (0.6%).
O’Keefe et al., 2025 [47]	AF	Meta-analysis of 8 RCTs (from 2013 to 2020) including 83,112 participants in various states of health	0.84–4 g/d EPA + DHA or EPA alone	2–7.4 y	Increased relative risk of developing AF (24%) (absolute risk: 4.0% vs. 3.3%; RR 1.24, 95% CI: 1.11–1.38). Daily doses ⁓1 g were associated with a 12% rise in AF risk, while doses from 1.8 to 4 g per day were linked to about 50% increase.
Tseng et al., 2025 [88]	Left ventricular ejection fraction (LVEF), peak oxygen consumption (VO_2_), blood B-type natriuretic peptide (BNP) concentrations, and quality of life	Meta-analysis of 14 RCTs (from 2006 to 2022) including 9,075 participants with HF	1–6.5 g EPA + DHA or EPA alone	4 wk – 6 y	Significant improvement in LVEF with high doses of EPA + DHA (2 to 4 g/day) and increase in peak VO2 with doses of ≥ 1 g/day (including those over 4 g/day) throughout ≥ 1 year. No effect on BNP or quality of life.

*EPA* eicosapentaenoic acid, *DHA* docosahexaenoic acid, *MI* myocardial infarction, *CVD* cardiovascular disease, *CHD* Coronary Heart Disease, *CAD* Coronary Artery Disease, *AF* atrial fibrilation, *HF* heart failure, *CI* confidence interval

Another meta-analysis of eight RCTs and published in 2025, involving a total of 83,112 participants examined the association between EPA and DHA and the onset of AF [47]. The findings indicated that supplementation with DHA and/or EPA was linked to a 24% higher relative risk of developing AF (absolute risk: 4.0% vs. 3.3%; RR: 1.24, 95% CI: 1.11–1.38; *p* = 0.0002). The increase in risk appeared to be dose-related: daily doses around 1 g were associated with a 12% rise in AF risk, while higher doses ranging from 1.8 to 4 g per day were linked to an approximately 50% increase.

In 2023, Huang et al. published a meta-analysis of 8 RCTs with 57,754 patients aimed to assess the effects of EPA and DHA supplementation on cardiovascular outcomes in patients with diabetes [86]. Six studies analyzed EPA + DHA at doses ranging from 0.85 to 4 g/day, whereas two trials tested EPA alone at doses of 1.8 or 3.6 g/day. While fixed-effects model analysis showed a significant reduction of cardiovascular events with these n-3 PUFAs (0.93; 95% CI: 0.90–0.97; *p* = 0.0009), subgroup analyses found no remarkable effects on the risk of fatal/nonfatal MI, major vascular events, CAD, AF, or all-cause mortality in patients with diabetes. However, subgroup analyses found that EPA, but not EPA + DHA, significantly reduced the risk of CVD in patients with diabetes (0.81; 95% CI: 0.73–0.90; *p* = 0.0001).

A meta-analysis published in 2024 included 18 RCTs with 134,144 participants in both primary and secondary cardiovascular prevention, investigating incident coronary revascularizations, MI, stroke, HF, unstable angina, and cardiovascular death [87]. Participants received either a combination of EPA + DHA (15 trials), EPA alone (3 trials), or a placebo/control, with follow-up periods ranging from 4.5 months to 7.4 years and the dose of DHA and/or EPA ranging from 0.6 to 5.4 g/day. Overall, EPA and DHA significantly reduced the risks of revascularization (0.90; 95% CI: 0.84–0.98; *p* = 0.001), MI (0.89; 95% CI: 0.81–0.98; *p* = 0.02), and cardiovascular death (0.92; 95% CI: 0.85–0.99; *p* = 0.02), with greater effects in studies where at least 60% of participants were on statins. Notably, EPA alone was more effective than combined EPA + DHA in reducing the risk of revascularization (0.76; 95% CI: 0.65–0.88; *p* = 0.0002) and showed greater benefit across all outcomes except HF.

A 2025 meta-analysis of 14 RCTs involving 9,075 participants evaluated the effect of EPA and DHA in patients with HF, focusing on dose and time-dependent effects [88]. The primary outcome measured was the change in left ventricular ejection fraction (LVEF), while secondary outcomes included changes in peak oxygen consumption (VO_2_), blood B-type natriuretic peptide (BNP) concentrations, and quality of life. This meta-analysis found that high doses of EPA + DHA (2 to 4 g/day) significantly improved LVEF, while EPA + DHA doses of ≥ 1 g/day (including those over 4 g/day) let to a remarkable increase in peak VO_2_ throughout ≥ 1 year compared to control. However, no significant differences were observed in BNP levels or quality of life between the n-3 fatty acid and control groups.

There has been some concern about bleeding risk with high dose EPA and DHA, as discussed elsewhere [3], although previous evaluations suggest no impact of high dose EPA and DHA on bleeding [89, 90]. A meta-analysis published in 2024 evaluated the bleeding risk associated with EPA and DHA and explored whether this risk was influenced by dosage or concurrent use of antiplatelet therapy [91]. The analysis included 11 RCTs encompassing 120,643 participants. All included studies assessed a combination of EPA and DHA, except for 2 studies with EPA only. Bleeding events, including fatal cases and those involving the central nervous system, were compared between the treatment and control groups. There was no significant difference in overall bleeding risk (RR: 1.09; 95% CI: 0.91–1.31; *p* = 0.34), nor were there notable differences in specific types of bleeding such as hemorrhagic stroke, intracranial or gastrointestinal bleeding. A predefined subgroup analysis included patients receiving high-dose purified EPA showed a 50% relative increase in bleeding risk, although the absolute risk rose by only 0.6%. The analysis also revealed that bleeding risk correlated with EPA dosage (risk difference 0.24; 95% CI: 0.05–0.43; *p* = 0.02), but not with the use of antiplatelet therapy.

## Final Discussion and Conclusions

As illustrated in the 2024 global n-3 index map [13], despite modest improvements in some regions, the overall global status of EPA and DHA remains suboptimal. Alongside well-established lipid-lowering and anti-inflammatory effects, recent research has uncovered other mechanisms of action of EPA and DHA that may influence cardiovascular health, including modulation of the gut-heart axis, regulation of immune-inflammatory responses, and effects mediated via ion channels and vagal tone. Findings from recent large-scale cohort studies have reinforced earlier evidence, consistently showing that higher intake or circulating levels of EPA + DHA are linked to a lower risk of developing CVD, especially CHD, having an MI and cardiovascular mortality in the general population. Furthermore, these studies suggest a protective effect against AF when EPA and DHA are consumed in moderate amounts (up to ~ 750 mg/day).

Although results from individual RCTs have been inconsistent, with REDUCE-IT and RESPECT-EPA demonstrating significant benefit, and STRENGTH, VITAL, OMEMI, OMEGA-REMODEL, yielding modest or neutral results, meta-analyses aggregating data from multiple RCTs generally support a significant reduction in major cardiovascular events. Notably, EPA monotherapy appears more effective than combined EPA + DHA formulations, as evidenced by trials such as REDUCE-IT showing significant benefits from EPA-ethyl ester and STRENGTH being terminated early due to futility. Emerging insights such as Bayesian reanalysis of VITAL as well as subgroup and responder analyses from other large cohorts, indicate that heterogeneity in outcomes may stem from multiple factors. These include differences in baseline cardiovascular risk, background statin therapy, dietary intake and bioavailability of EPA and DHA, and the relative potency of EPA vs. DHA in modulating lipid oxidation, inflammation, and plaque stability. Both individual biological characteristics (i.e. genetics, metabolism, baseline nutrient status) and study design features (i.e. dose, formulation, duration of follow-up, placebo choice, endpoint selection) can strongly influence the apparent efficacy of EPA and DHA interventions.

The foregoing discussion highlights that findings from large cohort and pooling studies are consistent in identifying an association between higher dietary intakes or blood levels of EPA and DHA and lower CVD risk and better CVD outcomes while RCTs are inconsistent in their findings, although meta-analyses of RCTs are generally consistent in demonstrating benefits. One distinction may be that cohort studies typically enrol a healthy population and monitor development of disease over a follow-up period and so are looking at prevention while RCTs often enrol those with pre-existing disease or at high risk because of the risk factor profile and so are looking at treatment. EPA and DHA may be more effective in preventing CVD than in treating it. The original findings of the VITAL trial and the recent re-evaluation of VITAL support that EPA and DHA have a role in prevention of CVD, especially in those who eat less fish and who have low EPA and DHA status and in some ethnic groups. Nevertheless, the inconsistent outcomes from cohort and pooling studies on one hand and RCTs on the other certainly deserve further consideration. Higher intakes and blood levels of EPA and DHA observed in cohort and pooling studies will be reflective of higher consumption of fish and/or use of supplements containing EPA and DHA. Whilst these lifestyle choices will certainly result in higher EPA and DHA exposure, this may be a surrogate for some other cardioprotective behavior. This may be a greater awareness of the importance of diet and lifestyle in maintaining health and the consequent adoption of a more healthy lifestyle including better dietary choices, more physical activity and better weight management. However, it is important to note that most, if not all, cohort studies have controlled for such factors in their statistical analysis, although there may always be some factors not accounted for (i.e. there may be residual confounding). Related to this, higher consumption of fish, so increasing EPA and DHA intake and blood levels, would be at the expense of another dietary component such as red meat, meaning that the higher EPA and DHA intake could be an indicator of removal of less healthy dietary components (saturated fatty acids, AA and so on). However, countering these possibilities, the findings of the VITAL RCT support that EPA and DHA themselves have a role in prevention of CVD. One other difference between cohort studies and RCTs is that the former study lifetime exposure, while the latter study a relatively short (in terms of life span) exposure in later life. It is evident that there needs to be better understanding of the reasons for differences between cohort and pooling studies of EPA and DHA and some of the RCTs with these fatty acids.

One emerging concern is the association between high dose EPA and DHA supplementation and AF, which has been shown across several RCTs and confirmed in meta-analyses [47]. Importantly, trials such as REDUCE-IT which used 3.6 g of pure EPA and RESPECT-EPA, which used 1.8 g/day of pure EPA, reported an increased risk of AF alongside significant reductions in major cardiovascular events [72, 80]. Additionally, OMEMI which tested 1.6 g of EPA + DHA also observed an elevated AF risk but did not demonstrate cardiovascular benefit [77]. Meta-analyses integrating these and other studies indicate a dose-dependent relationship with the risk of incident AF rising as the daily dose of EPA and DHA increases above 1 g/day [92]. Understanding the mechanism behind this pro-arrhythmic effect is important for identifying at-risk individuals and guiding safe use. Finally, most studies show minimal risk of bleeding associated with EPA + DHA [89–91]. While high-dose EPA monotherapy may slightly increase bleeding risk, its clinical relevance is generally considered low.

RCTs of DHA and/or EPA have highlighted a number of areas for future research. The most obvious are whether EPA and DHA have different clinical impacts and, if so, why that is; the identification of the most effective dosing of these fatty acids; whether different sub-groups would benefit more from these fatty acids than others; and whether AF is a true risk of high dose EPA or EPA + DHA intervention. Evaluation of biomarkers indicates overlapping effects of EPA and DHA, although there are different impacts on LDL-cholesterol and on HDL sub-classes and different efficacies with regard to TG lowering and inflammation [20] while the different findings of REDUCE-IT and STRENGTH have led to questions about the interaction of EPA and DHA. Related to this is the question of dose: effects on biomarkers and clinical outcomes are clearly dose-dependent but there is no agreement on the most optimal dose for either prevention or treatment of CVD. One complication of high dosing may be adverse impacts such as AF and whether this is a real possibility needs further investigation. Finally, trials such as VITAL suggest sub-groups who might benefit more from EPA + DHA and this merits more research. Certainly those with low intakes and low blood levels are likely to benefit more from EPA and DHA than those with higher intakes and blood levels, but further investigation of the influence of ethnicity, genetics (e.g. polymorphisms in genes related to cardiovascular risk), body fatness, age, sex, medications and other dietary components on how effectively EPA and DHA prevent and treat CVD is warranted. Such research should pave the way for more personalized EPA + DHA advice and therapy and may also help to resolve existing inconsistencies in the literature.

In conclusion, EPA and DHA act through multiple mechanisms suggesting that they have a role in prevention and treatment of CVD. Cohort and pooling studies and some RCTs support their role in prevention, but RCTs of therapeutic effects demonstrate inconsistent findings. Effects of EPA and DHA are dose-dependent and most likely influenced by other factors. The inconsistencies in the findings of RCTs provide directions for new research in order to more fully understand the therapeutic benefits of EPA and DHA.

## Data Availability

No datasets were generated or analysed during the current study.
